# Future of Using Robotic and Artificial Intelligence in Implant Dentistry

**DOI:** 10.7759/cureus.43209

**Published:** 2023-08-09

**Authors:** Abdullah Saeed, Mohammed Alkhurays, Mohammed AlMutlaqah, Mohammed AlAzbah, Sarah A Alajlan

**Affiliations:** 1 Department of Research, Ministry of Health, Abha, SAU; 2 Department of Prosthodontics, Dental Center, Abha, SAU; 3 Department of Prosthodontics, Ministry of Health, Abha, SAU; 4 Department of Dental Health, Ministry of Health, Abha, SAU; 5 Clinical Pharmacy, Almaarefa University, Riyadh, SAU

**Keywords:** ai and robotics in healthcare, healthcare, dentist, implant dentistry, artificial intelligence

## Abstract

The integration of robotic technology and artificial intelligence (AI) in implant dentistry has ushered in a new era of precision and efficiency. This abstract aims to understand the integration, implications, potential, and challenges of robotic technology and AI in implant dentistry. Robotic systems offer unparalleled accuracy in implant placement, reducing human error and improving treatment outcomes. AI algorithms analyze extensive patient data to assist in diagnosis, treatment planning, and implant design, optimizing the overall process. Successful case studies demonstrate improved implant survival rates and patient satisfaction. However, ethical considerations and the balance between human expertise and reliance on technology must be addressed. Ongoing research aims to enhance these technologies and integrate them with digital workflows. Collaboration and knowledge sharing among practitioners, researchers, and industry experts are essential to drive progress and ensure responsible implementation. The future of implant dentistry lies in harnessing the potential of robotics and AI while upholding the highest standards of patient care and ethical practice.

## Editorial

The field of dentistry has witnessed tremendous advancements in recent years, owing to the integration of robotic technology and artificial intelligence (AI). The marriage of these two cutting-edge domains has ushered in a new era in implant dentistry, revolutionizing the way we approach oral healthcare. In this editorial letter, we delve into the fascinating world of robotic and AI-assisted implant dentistry, exploring its potential, challenges, and implications for both patients and practitioners. The use of robotics and AI in implant dentistry holds immense promise for enhancing the precision, efficiency, and overall success of dental implant procedures. Robots equipped with advanced algorithms and sensors are capable of performing intricate tasks with unparalleled accuracy, ensuring optimal placement of implants and minimizing the margin for error. By reducing human error, these technologies have the potential to improve treatment outcomes and patient satisfaction, paving the way for a more predictable and streamlined implant process [1]. Furthermore, the integration of AI into implant dentistry brings forth numerous benefits. Machine learning algorithms can analyze vast amounts of data, allowing dentists to make evidence-based decisions and tailor treatment plans to an individual patient's needs. AI systems can assist in diagnostic procedures, aid in the planning and design of implants, and even provide real-time feedback during surgery, enabling clinicians to make informed decisions in real time.

However, as with any disruptive innovation, there are challenges and ethical considerations that must be addressed. The role of human expertise and judgment in conjunction with robotic and AI technologies is a critical aspect that requires careful consideration. Dentists must strike a balance between leveraging the advantages of these advancements and maintaining their professional autonomy and responsibility. In addition, ethical concerns surrounding patient privacy, data security, and potential biases in AI algorithms necessitate ongoing scrutiny and regulatory frameworks [2]. 

In this editorial, we explore the current state of robotic and AI-assisted implant dentistry, shedding light on the most recent developments, successful case studies, and ongoing research. We aim to provide a comprehensive overview of the benefits, challenges, and ethical considerations associated with these technologies and encourage a thoughtful and informed dialogue among practitioners, researchers, an industry experts. As the field of implant dentistry continues to evolve, it is essential for dental professionals to stay informed about the latest advancements and embrace the potential of robotics and AI. By harnessing the power of these technologies responsibly, we can enhance patient care, expand treatment options, and ultimately transform the landscape of implant dentistry [3]. The current state of robotic and AI-assisted implant dentistry is rapidly evolving, offering exciting possibilities for improved patient outcomes and enhanced dental care. Recent developments in this field have showcased the potential of these technologies, and numerous successful case studies have demonstrated their effectiveness. Furthermore, ongoing research continues to explore and refine the applications of robotics and AI in implant dentistry. One of the key benefits of robotic technology in implant dentistry lies in its precision and accuracy. Robotic systems, equipped with advanced sensors and algorithms, can assist dentists in achieving optimal implant placement. These robots can navigate anatomical structures with exceptional accuracy, reducing the risk of errors and complications.

By ensuring precise positioning of implants, robotic technology contributes to the long-term success and stability of dental restorations [4]. Meanwhile, AI plays a crucial role in data analysis and decision-making. Machine learning algorithms can process vast amounts of patient data, including medical history, diagnostic imaging, and treatment outcomes, to provide valuable insights and assist dentists in formulating personalized treatment plans. AI algorithms can identify patterns, predict potential complications, and recommend the most appropriate implant designs, optimizing the overall treatment process. Several successful case studies have demonstrated the effectiveness of robotic and AI-assisted implant dentistry. For example, robotic systems have been used to perform complex implant surgeries with a high degree of precision, leading to improved implant survival rates and patient satisfaction. AI algorithms have aided in the accurate identification of bone quality and quantity, facilitating optimal implant placement and reducing the risk of implant failure. These advancements have allowed for shorter surgery times, improved aesthetic outcomes, and enhanced patient comfort [5]. 

Despite the numerous benefits, there are also challenges and ethical considerations that come with the integration of robotics and AI in implant dentistry. One of the primary concerns is the proper balance between human expertise and reliance on technology. Dentists must maintain their clinical judgment and ensure that technology is used as a supportive tool rather than replacing their professional judgment. In addition, ethical considerations include patient privacy, data security, and potential biases in AI algorithms. It is crucial to address these concerns through regulatory frameworks and ongoing monitoring of these technologies. Ongoing research in robotic and AI-assisted implant dentistry focuses on expanding the capabilities of these technologies. This includes the development of more sophisticated robotic systems that can adapt to complex anatomical variations and improved AI algorithms that can analyze multi-modal patient data for more accurate treatment planning.

In addition, research is being conducted to enhance the integration of robotic and AI technologies with digital dentistry workflows, such as computer-aided design and manufacturing (CAD/CAM) systems, to streamline the entire implant process. To encourage a thoughtful and informed dialogue among practitioners, researchers, and industry experts, it is important to foster collaboration and knowledge sharing. Professional organizations and academic institutions can play a vital role in facilitating conferences, workshops, and forums dedicated to discussing the advancements, challenges, and ethical considerations in robotic and AI-assisted implant dentistry. These platforms allow experts to exchange ideas, share experiences, and collectively work toward refining these technologies for the benefit of patients worldwide.

The integration of robotic technology and AI into implant dentistry has drastically reshaped the field, offering unprecedented strides in oral healthcare. This in-depth editorial focuses on robotic and AI-assisted implant dentistry, investigating its potential, hurdles, and implications for both practitioners and patients alike. Robots, armed with sophisticated algorithms and sensors, undertake intricate tasks with unmatched precision, optimizing implant placement and significantly reducing errors. This level of accuracy leads to improved treatment outcomes and elevated patient satisfaction, providing a more predictable and efficient implant process. In tandem with robotics, AI brings an array of benefits to implant dentistry. Machine learning algorithms can analyze large-scale data, facilitating evidence-based decisions and customizing treatment plans to an individual patient's unique needs. AI can assist in diagnostics, planning, and implant design and even provide real-time feedback during surgeries.

The integration of robotics and AI in implant dentistry represents a groundbreaking shift in oral healthcare. Robots, equipped with precise algorithms, optimize implant placements, paving the way for improved longevity and effectiveness of dental prostheses. AI complements this by processing vast datasets, enabling evidence-based, individualized treatment plans and providing invaluable assistance from diagnostics to surgical feedback. However, the financial implications of incorporating these technologies cannot be overlooked. While initial investments for dental practices are significant, potential long-term benefits, such as reduced surgical times, and enhanced patient outcomes might offer economic advantages. However, amid technological advancements, the irreplaceable human element in dentistry remains paramount. While robots offer precision and AI contributes analytical prowess, the human connection, intuition, and empathy intrinsic to successful patient-practitioner relationships are non-negotiable. Ethical considerations, especially concerning patient privacy, data security, and potential AI biases, demand vigilant scrutiny. For instance, ensuring that AI does not perpetuate biases from limited training datasets is crucial. As the realm of implant dentistry evolves, collaborative endeavors across practitioners, researchers, and industry leaders are essential to harness the full potential of these innovations responsibly, always prioritizing patient outcomes.

However, alongside these revolutionary advances, several critical questions arise. How can we ensure that AI and robotic technologies supplement rather than replace human expertise? How can dental professionals strike the right balance between leveraging these advancements while maintaining their professional autonomy and responsibility? How do we address ethical concerns surrounding patient privacy, data security, and potential biases inherent in AI algorithms? These questions underscore the necessity for ongoing oversight and robust regulatory frameworks. As we delve deeper into the state of robotic and AI-assisted implant dentistry, we aim to illuminate these aspects and offer informed discourse among practitioners, researchers, and industry experts.

Ongoing research is looking into refining these technologies. Focus areas include sophisticated robotic systems adaptable to complex anatomical variations and advanced AI algorithms capable of multimodal patient data analysis for more precise treatment planning. Further integration of these technologies with digital dentistry workflows, such as CAD/CAM systems, is another active research area.

To the readers, we encourage you to remain abreast of these developments and participate in continuous learning. Attend conferences, seminars, and workshops on the subject, and consider contributing your insights, experiences, and ideas to the academic and professional discourse. Understand the ethical considerations associated with these technologies and adopt a responsible approach to their implementation.

In conclusion, while the integration of robotics and AI in implant dentistry presents substantial potential, it also calls for the responsible addressing of the challenges and ethical considerations. By fostering dialogue and collaboration, we can responsibly harness these technologies' benefits while upholding the highest standards of patient care and ethical practice. We hope this editorial sparks further exploration and discussion, propelling the field forward while ensuring high standards of care for patients. Together, let us navigate this exciting frontier of dentistry, where technology meets human expertise, to create a promising future.
